# Adaptive Dynamics of Regulatory Networks: Size Matters

**DOI:** 10.1186/1687-4153-2009-618502

**Published:** 2009-02-03

**Authors:** Dirk Repsilber, Thomas Martinetz, Mats Björklund

**Affiliations:** 1 Department of Genetics and Biometry, Research Institute for the Biology of Farm Animals (FBN), Wilhelm-Stahl Allee 2, D 18196 Dummerstorf, Germany; 2 Institute for Neuro- and Bioinformatics, University of Lübeck, Ratzeburger Allee 160, D 23538 Lübeck, Germany; 3 Department of Animal Ecology, Evolutionary Biology Centre, University of Uppsala, Norbyvägen 18 C, 75236 Uppsala, Sweden

## Abstract

To accomplish adaptability, all living organisms are constructed of regulatory networks on different levels which are capable to differentially respond to a variety of environmental inputs. Structure of regulatory networks determines their phenotypical plasticity, that is, the degree of detail and appropriateness of regulatory replies to environmental or developmental challenges. This regulatory network structure is encoded within the genotype. Our conceptual simulation study investigates how network structure constrains the evolution of networks and their adaptive abilities. The focus is on the structural parameter *network size*. We show that small regulatory networks adapt fast, but not as good as larger networks in the longer perspective. Selection leads to an optimal network size dependent on heterogeneity of the environment and time pressure of adaptation. Optimal mutation rates are higher for smaller networks. We put special emphasis on discussing our simulation results on the background of functional observations from experimental and evolutionary biology.

## 1. Introduction

The organic world—from a system's biological point of view—could be understood as organized in interacting networks at all possible organisational levels. Each organisational level contains interacting units. The forms and patterns of interaction among such units vary considerably both in time [1] and across different biological taxa [2]. It is increasingly accepted that adaptability and robustness are inherent *network properties*, and not a result of the fine tuning of single components' characteristics [3–5]. Interacting networks can, for example, be found at the molecular genetic level where genes and their products interact to enhance or suppress the effect of each other, a pattern collectively termed epistasis. At this level of genotype-phenotype mapping, interactions are the rule rather than the exception. The concerted action of genes and their products creates the phenotypes we observe.

Research on structural properties of regulatory networks, especially for gene regulatory networks in a developmental context, has long been focused on *internal* structural properties [6–8], for reviews see [9] or [10]. This does not take into account environmental changes, nor is it intended to consider evolutionary aspects. The situation has changed recently as [11–13] studied evolutionary performance of simulated regulatory networks with their focus on network structures with different connectivities. Also, studies on optimisation from a computational and more technical motivated perspective regarding the interactions of evolution and phenotypic plasticity have become available [14, 15].

However, these approaches did not take into account the *size* of regulatory networks and its relevance for evolutionary dynamics and phenotypic plasticity, that is, biological function. Network size can either be understood as referring to *genome size* or to the *size of regulatory modules* which are the building blocks of the entire regulatory system, either at the cellular level [16–18], or at the level of integration of different parts of the organism [19]. This led to our contribution of a *conceptual* comparative study with the *focus on network size*.

Concerning the size, two kinds of regulatory networks can be identified, being at opposite ends of a continuum. On the one side, we have the smallest network possible with two interacting units, and on the other side we have an infinite number of interacting units with an infinite number of interactions. There are some general properties of these networks that deserve attention and help to understand why small networks are favored by selection in some cases, and why larger networks are favored in other cases.

Small networks have three main features: they can cope only with a limited small number of environmental challenges. Therefore, within a heterogeneous environment this limitation of detail in response enables only a limited adaptedness. Secondly, evolution needs only a few steps to change a small network's structure and its repertoire of responses. Thirdly, small networks are cheap to run and maintain. Large networks on the other hand can cope with many different tasks. Due to their large repertoire and the resulting possibility of detailed adaptive responses they enable higher adaptedness in a heterogeneous environment. However, large networks are both slow in terms of evolutionary change as well as costly to run and maintain. Hence, regarding their abilities enabling adaptedness and evolutionary change, small and large regulatory networks are at opposite sites of the classical "stability-flexibility dilemma" [20].

In this contribution, we want to pose the question whether there are general properties regarding phenotypic plasticity and evolutionary dynamics for regulatory networks of different size. We refer to Thoday who already in 1953 stated that 

" a heterogeneous or unstable habitat will lead to selection for variability; this may result in a flexible genetic system or a flexible developmental system or both. The more flexible the developmental system, the less flexible the genetic system need be, and the strength of selection for the two types of flexibility must depend largely upon the relations between generation time, the rate of environmental change, and the heterogeneity of the environment." [20]

To stress the biological meaning of "flexibility," we use instead the concept of adaptation, adaptability, and adaptedness [21]. Here, adaptation refers to a specific response of a system to an external challenge. Adaptedness characterises the appropriateness of an adaptation, or of the number of adaptations a regulatory system can realise. Adaptability refers to the—structurally based—ability of a regulatory system to be or become adapted to a number of different challenges in a changing environment. Adaptability in our context, thus, is realized on both the level of phenotypic plasticity and evolutionary optimisation.

In our study, we investigate evolutionary adaptability of regulatory networks as a function of their size, that is, a network structural constraint. We address this question taking a conceptual modeling approach. Evolutionary dynamics of simulated regulatory networks of different sizes were evaluated in relation to the heterogeneity of tasks to be performed. Here, a more biologically oriented reader might think of different habitats, or temporally changing environmental conditions. We simulate the evolution of a population of networks which compete in terms of relative fitness. Fitness is understood as probability of leaving descendants as in [20]. Regarding evolutionary dynamics, the interesting level is the level of the phenotype, since this is the level selection acts on. Differences in gene-gene interactions are visible to selection and further evolution only if they translate into phenotypical differences among individuals. We take a very simplistic approach to explicitly modeling this *genotype-phenotype map* and employ a parsimonious model by using the Steinbuch network model [22].

This model choice is also based on a major result of statistical network modeling. Analyses of distributions of simple regulatory motifs both in prokaryotes and in eukaryotes point to similar results; the so-called *multi-input motif* is a significant and prominent part of regulatory biological networks [23–25]. It is a two-layer feed-forward network. The information about which input vector leads to which output vector (response) is encoded within the pattern of presence/absence of connections between these two layers. We are going to use this approach as a conceptual model for regulatory networks. We introduce mutations that change both wiring and size of the network and discuss the possibility of an optimal network size.

Within the discussion, we devote special emphasis on four examples for observations of natural evolution where the size of the underlying regulatory networks—and their evolutionary dynamics as well as characteristics of adaptability—may play a decisive role.

## 2. Methods and Model

As we investigate network structural impacts on two different kinds of adaptive processes, evolutionary adaptation and phenotypic plasticity, our simulation setting includes evolution of network encoding genotypes (individuals) as well as evaluations of the regulatory replies of these individual networks to environmental challenges.

### 2.1. Individual Genotypes

**Figure 1:**
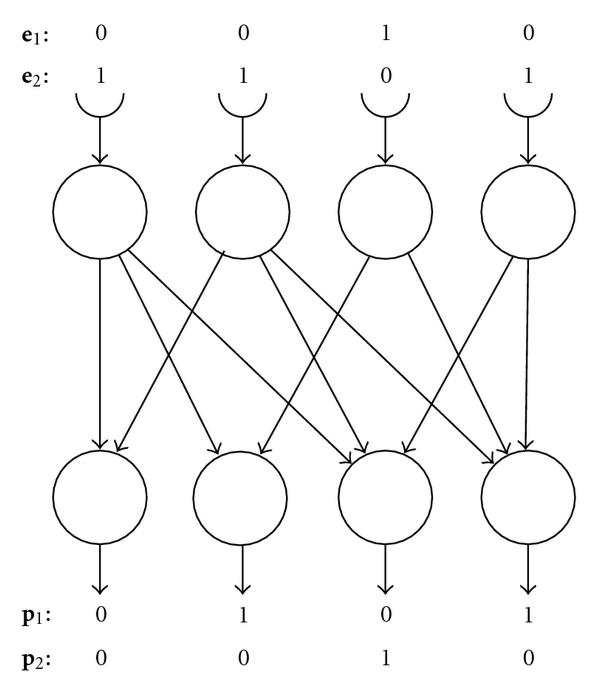
**Scheme for the "4-net" () with two examples for environmental input () and corresponding responses ().** This input-output function can be modeled as a simple matrix multiplication combined with maximum thresholding (see (3)).

Each individual in the model population is a simulated regulatory network of the Steinbuch matrix type [22], which is a two-layer feed-forward threshold network with  nodes in both input and output layers. It is structurally equivalent to the multi-input motif as illustrated in Figure 1. Each entry in such an  matrix , with  for , has two possible states, 0 or 1. Consider an example for  with , where the dimension  is also referred to as *network size*:(1)(2)

For  there is a connection from input layer node  to output layer node  (see Figure 1), for  there is no connection.

In this manner, the genotype  of each individual specifies the regulatory interactions within its regulatory network, that is, its *network structure*.  which is chosen as in (1) represents the regulatory network illustrated in Figure 1. During simulated evolution, matrix  is represented as a linearized genotype *vector, genes*, as exemplified in (2).

### 2.2. Modelling The Environment

Environmental challenges are modeled as -dimensional column vectors,  with , such that  can take values either 1 or 0.  is the input for node  of the input layer (cf. Figure 1). Environmental heterogeneity is accounted for by the number of different environmental challenges presented for the individual regulatory network during a single generation run.

### 2.3. Modelling Phenotypes

The phenotype of each individual is also modeled as -dimensional column vector:  with , and is determined from genotype  and environment  as (3)

for , being a special case of a threshold feed-forward network. The thresholding in our model is a maximum threshold, such that, all genes of an individual together determine the structure of the genotype-phenotype map, which combines genotype  and environment  into the resulting phenotype vector .

For illustration consider two examples of environmental inputs,  and : (4)

To determine the belonging phenotypes we multiply with  and apply the thresholding as indicated: (5)

For  and  compare Figure 1.

Environmental heterogeneity  was modeled by presenting more than one environmental input per generation to the network as discussed in Smolen et al. [9], for example, to model an environmental heterogeneity of , eight different randomly generated inputs were chosen. Probabilities of entries "0" or "1" were 50% each. These environmental inputs were then applied to each network in each generation of the simulation run. This means that environmental challenges remain unchanged during the evolution simulated in a single simulation run. For the next simulation run, new environmental vectors were randomly generated, with their number according to the environmental heterogeneity chosen.

### 2.4. Fitness

For each environment  and environmental heterogeneity , with , an *a-priory* optimal phenotype  has been fixed before the simulation. The elements of  are drawn at random with probabilities (6)

prior to the respective simulation runs.

The fitness of each individual  was calculated as one minus the mean value over the Hamming distances between actual and optimal phenotypes for each environmental condition, indicating how well the actual phenotype matches the *a priori* given optimal phenotype:(7)

### 2.5. Evolution

We used a strict truncation selection and only kept the individuals with the highest fitness. Mutation rates were between  per generation per gene and recombination rates between  per generation per genome.

Simulations were run either with *fixed* or with *variable* network size . Runs with variable network size started either with a uniform distribution of network sizes  or with small networks throughout, and allowed for changing the network size within this range with probability . Individuals were modeled to encode a specific genotype  by using a linearized vector *genes* with the entries  of  and length  (see (2)). In simulations with variable network size, a genotype vector for a given individual could be elongated from the existing  entries to  entries, corresponding to the next larger network size , or also shrinked to length  by deleting the  last elements, leading to the network of network size .

### 2.6. Simulated Scenarios

For runs with *fixed* network size we used (8)

For runs with *variable* network size we used (9)

In summary, the key parameters of variation were network size , the environmental heterogeneity  and the mutation rate . All simulations were implemented in C using the LibGA package [26].

**Figure 2:**
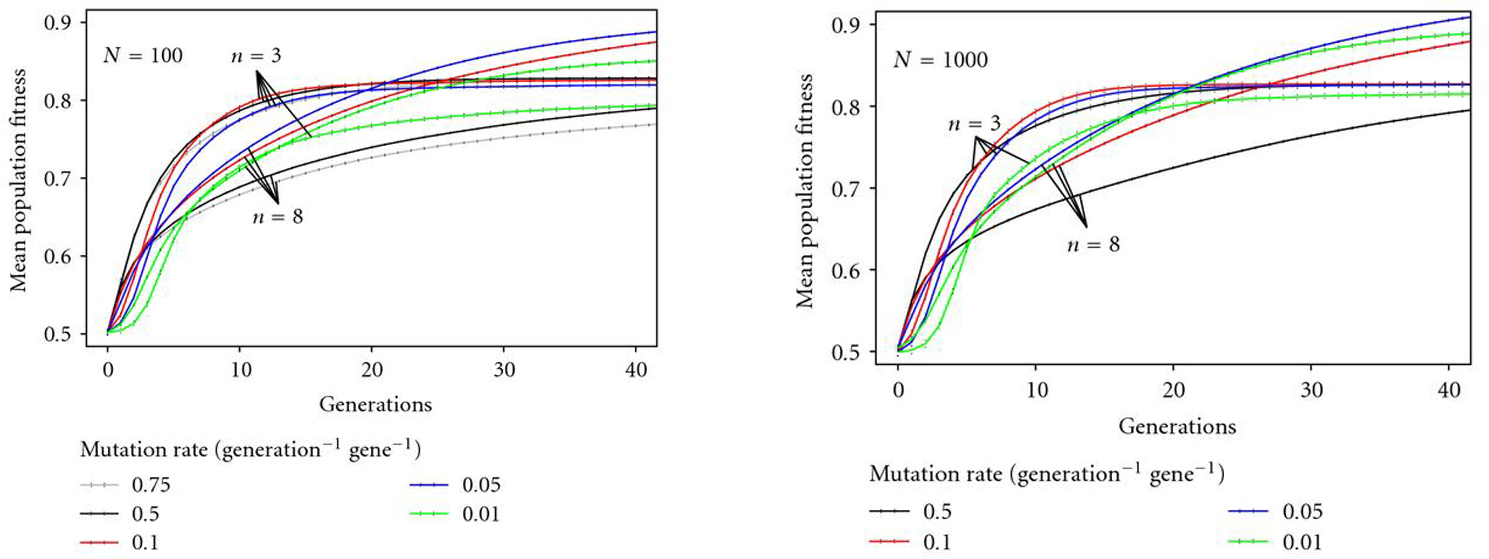
**Adaptation dynamics for population sizes of  and  for different mutation rates; optimal mutation rate depends on network size.** The dynamics of mean population fitness between 1 and 40 generations are shown. Solid lines depict the mean-value over 2000 repetitions, whereas dotted lines give the standard errors of the population means.

## 3. Results

The questions guiding our investigations, regarding the evolution of populations of networks with *fixednetwork size*, were the following. 

(1)Does network size influence evolutionary dynamics?

(2)Does network size influence optimal mutation rate with respect to higher maximum fitness?

(3)Does recombination rates have a relevant influence regarding these questions?

Regarding the evolution of populations of networks with *varying* network size we asked the following questions. 

(4)Does the distribution of network sizes in a population change during evolution?

(5)Is there an optimal network size for a given environmental heterogeneity?

**Figure 3:**
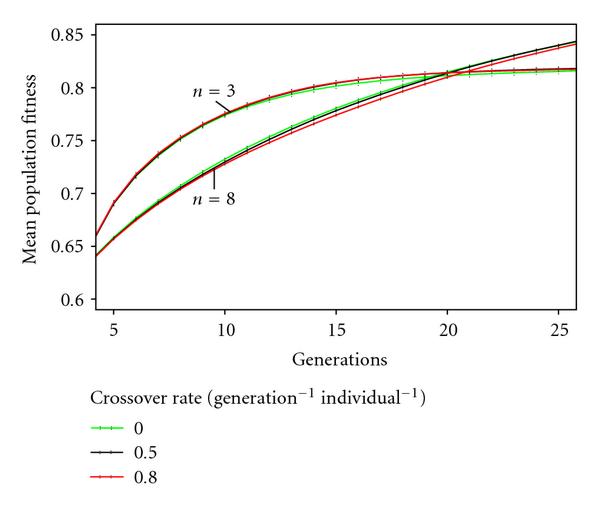
Comparison of adaptation dynamics for different recombination rates. Adaptation dynamics were simulated for 25 generations, 2000 runs each, using a mutation rate of . No significant impact of the cross-over rate can be seen, also if compared to Figure 2 (for ).

**Figure 4:**
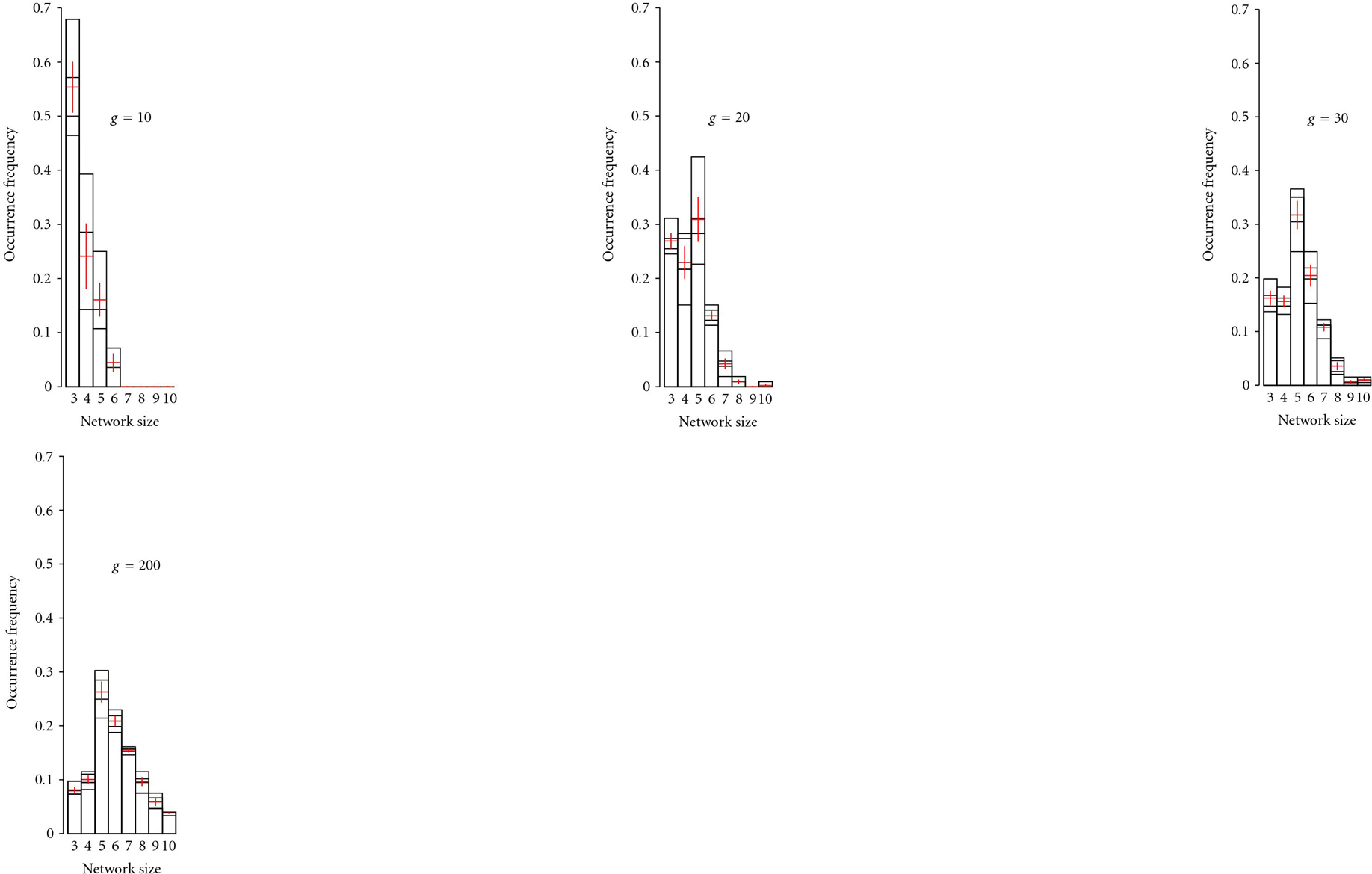
**Distribution of network size as a function of time for adaptation.** The distributions of network-size were calculated from 4 repetitions of 2000 runs each, for adaptation times of 10, 20, 30, 200 generations, and an environmental heterogeneity of . Most prevalent network-size increases with time for adaptation.

**Figure 5:**
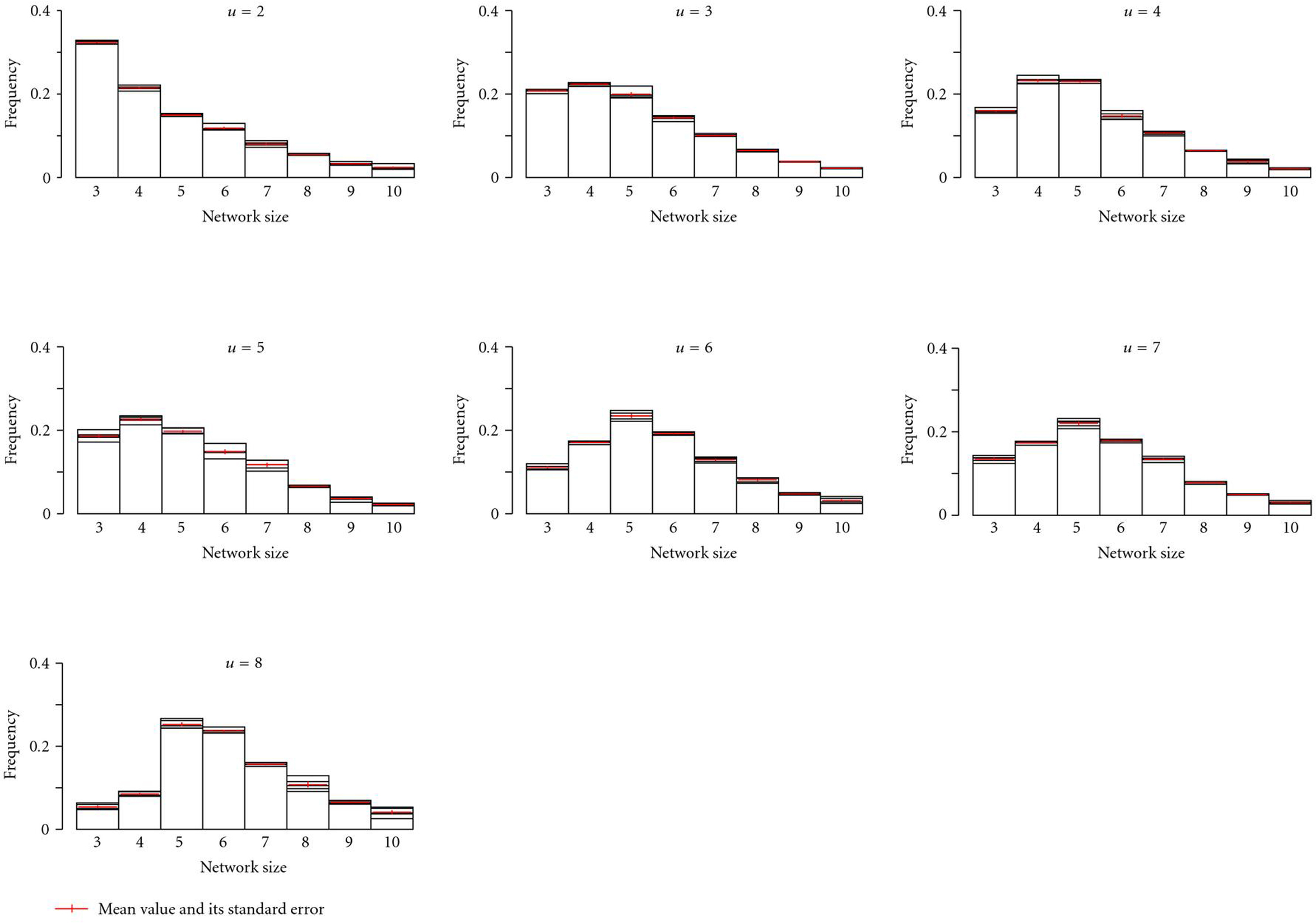
**Distribution of regulatory network sizes as dependent on environmental heterogeneity.** Optimal regulatory network size is increasing with environmental heterogeneity. Mean values and their standard errors over 5 repeated experiments are shown, where each experiment covered 5000 simulation runs (5000 generations for each simulation run).

Generally, during simulations each single population reached a different mean fitness. Therefore, we used mean values over populations as characterisation of the population dynamics. Regarding our questions, simulations resulted in the following. 

(Re 1)Adaptation dynamics for a population of networks of different size (, ) revealed that small networks reached a higher average fitness as compared to large networks at five generations (Figure 2). However, as time proceeds, large networks reached a higher average fitness than the small ones for most mutation rates after around 20 generations. This pattern was the same for both population sizes  and .

(Re 2)The optimal mutation rate is dependent on network size; for the small network a mutation rate of  resulted in the largest maximum average fitness, whereas for the larger network the optimal mutation rate was lower ().

(Re 3)Size and recombination rates do not interact; recombination rates did not affect previous results significantly (Figure 3). Therefore, we used a recombination rate of  throughout our further experiments.

(Re 4)Simulation runs were started with small () networks and mutable network size. After 10 generations networks of size 3 were most common, but network size increased rapidly so that after 30 generations networks of size 5 were most common in the population (Figure 4). After 200 generations networks of size 5 were still the most common ones, but the largest networks () increased in frequency and the smaller ones decreased in frequency (Figure 4).

(Re 5)We tested whether there are *optimal* network sizes for a given environmental heterogeneity. To evaluate this, we started the simulations with equally distributed networks sizes  and recorded network sizes after 5000 generations for different levels of environmental heterogeneity (, , numbers of runs  = 5000). Smaller networks were favored at low levels of  (Figure 5), but optimal network size increased with . However, this increase was not linear so that  was the optimal for most of the higher levels of environmental heterogeneity ().

## 4. Discussion

In our conceptual simulation study we have investigated the relation of a specific structural parameter of regulatory networks, *network size*, to their functional abilities, *phenotypical adaptability*, and *evolutionary dynamics*. We used Steinbuch matrix models to explicitly model the genotype-phenotype mapping in regulatory networks, evolving in silico under different environmental heterogeneities. Our investigation aims at contributing to an understanding of different kinds of adaptive pressures for different niches, and thus providing insights what to look for in general properties of regulatory networks. This could serve as a starting point for a quantitative or predictive treatment of such phenomena.

Results show that *time pressure of adaptation* and *environmental heterogeneity* clearly interact when favoring either small or large regulatory networks during evolution—as can directly be inferred from Figures 2 and 5 (our objectives (1) and (5)).

**Figure 6:**
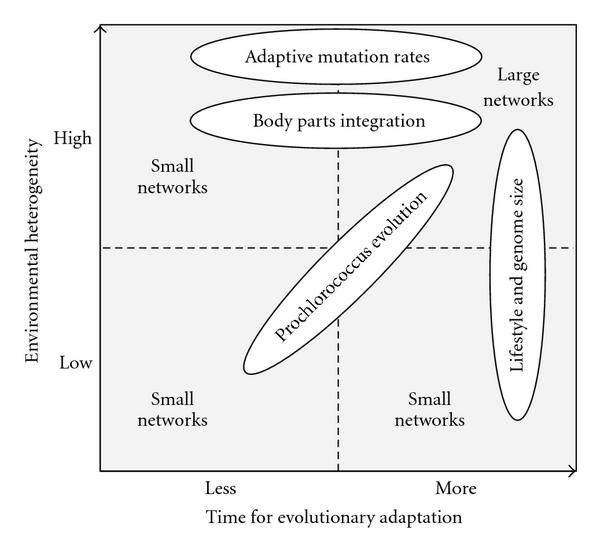
**Dependency of network size prevailing during evolution as dependent on environmental heterogeneity and time pressure of adaptation—exemplified by the four biological examples discussed.** For each example, this scheme illustrates the different reasons for small and large network sizes observed.

For relatively stable environments, small network size is favored both for shorter as well as for a longer time scale of the evolutionary process. However, in heterogeneous environments, smaller networks have an evolutionary advantage only over short time-scales, while larger networks gain an advantage over longer time scales. To illustrate these main results, Figure 6 shows the interaction of factors *time pressure of adaptation* and *environmental heterogeneity* resulting in prevalence of either smaller or larger networks.

In addition, our simulations show that in heterogeneous environments average fitness does not increase monotonically with network size. Rather, there seems to exist an *optimal network size* given the level of environmental heterogeneity. Also, larger regulatory networks were dependent on modest mutation rates for reaching maximum adaptedness. Recombination rate and size of simulated populations were not relevant for these results.

In the following we are shortly discussing possible reasons for the observed results in our simulation study and then focus on four biological examples, where we propose that the phenomena observed can be linked to evolutionary implications of network size.

It may be argued that in heterogeneous environments a large regulatory network may *always* be advantageous since it can respond to multiple environmental inputs with the most differentiated response possible, that is, a high degree of plasticity. Thus, a large network can be assumed to be able to differentiate more correctly between a large number of environmental differences, and thus respond in the most optimal way to each of the environmental challenges, leading to high adaptedness. However, the results presented here clearly point to an additional important factor determining evolving regulatory networks sizes; time pressure of adaptation. In the examples following, it will become apparent that this time pressure can either result by the observer—that is, by setting a deadline for adaptation from an *outside* observing schedule—or by competition, that is, by a system *inherent* factor. Time pressure of adaptation has three consequences for the network size reached by an evolutionary process.

First, small networks are evolving faster due to a reduced search space, an important factor which is obvious from a statistical model fitting and optimisation perspective. Second, epistasis effects are reduced, while in a large redundant network mutations may be masked. Third, consider observing the evolving system after a really long time—such that there does not seem to exist any time pressure of adaptation any longer. However, there are medium large-scale networks existing within the evolving population which are showing already near-to-perfect adaptedness. Under these circumstances networks of still larger size are very unlikely to evolve as they need to show a clearly improved fitness already from the beginning to be able to compete. It is also arguable if such long periods without time pressure of adaptation exist at all. If, however, some environmental event would cause further increase in environmental heterogeneity—our results would propose to expect a further evolutionary growth of the responsible regulatory networks.

Now consider four biological evolving systems, which we propose to exemplify the interaction of environmental heterogeneity and time pressure of adaptation as major determinant of the favored network size.

### 4.1. Microbial Genome Size and Life Style

In our model, prevailing sizes of regulatory networks are dependent on environmental heterogeneity. Our results predict that levels of epistatic interactions and size of linkage groups should be low in populations adapted to capricious environments both on shorter as well as on longer time scales of evolution. This may give a hint to explain the notion that genome size seems to be lifestyle dependent in microbial organisms (see e.g., [27] for a review, or else [28–32]). Either direction of change in genome size is thought of being dependent on heterogeneities in the living conditions; on the one hand, the constant environment of intracellular parasites renders numerous genes expendable, leading to usually irreversible gene loss. On the other, changes in habitat to a more complex environment seem to lead to the contrary effects. As Stêpkowski and Legocki [27] point out in their review, there seems to be a "need for a great number of capabilities" to accomplish adaptation to changing environmental conditions, which is met by integrating numerous genes. Our model predicts overrepresentation of larger regulatory networks in more heterogeneous environments, while for stable environments a small regulatory networks dominate (see results illustrated in Figure 5, as well as overview in Figure 6).

### 4.2. *E. coli* Mutation Rates in The Mouse Gut

Our findings may also add a new viewpoint to the ongoing "adaptive mutability" debate. Giraud et al. [33] discuss their findings of elevated mutation rates in the beginning of the invasion of inoculated mice guts with *E. coli* strains as "adaptive mutability." However, also in our model, using a *constant* mutation rate, small networks dominate the first stage of adaptation, whereas changes to larger networks occur in the longer perspective (see Figure 4). As far as the mutation rates are concerned, we found that higher mutation rates are leading to higher adaptedness of small regulatory networks, whereas lower mutation rates are favoring higher adaptedness in the evolution of larger regulatory networks (see Section 3 (Re 2)). We therefore propose the following hypothesis to explain the findings of Giraud et al.: our simulation results would suggest that, during the early phase of adaptation to the mouse gut the bacteria adapt in coarse-grained, larger steps due to changes in small regulatory networks. Together with our simulation results which showed that *high* mutation rates favor better adaptation in small networks, it becomes likely that the observable result of evolution during the early phase will show a mutation record leading to a *high* estimated mutation rate.

In the later phase, as for Figure 4, when it comes to fine tuning the system to conditions in the mouse gut, an increase in adaptedness has to rely on large regulatory networks. As these are capable to realise a larger number of adaptations, they have to be the basis for an adaptation to a more detailed perception of the new environment. As for the early phase, the observer will account for only the results of selection. This time however, as for large networks a lower mutation rate is favorable, selected networks will show a mutation record leading to estimate a lower mutation rate.

Central for this hypothesis is the idea that the small regulatory networks, which are responsible for early evolutionary adaptation, are *not identical* with the larger regulatory modules optimized in the longer timescale. Summarising our hypothesis, observed "adaptive mutabilities" could be explained as a product of ongoing selection in an adaptation process under the constraints of the adapting regulatory systems.

### 4.3. Selection of Correlated Traits: Body Plans and Size

The level of integration of body parts in plants and animals is mainly caused by pleiotropy. It can be shown that the level of genetic correlation among different parts of an organism largely determines the evolutionary response to selection [19, 34, 35]. For example, a large number of *highly correlated* traits of an organism—corresponding to *large* regulatory networks in the frame of our simulation model—almost invariably lead to a response in terms of overall body size, even though the pattern of selection might be, for example, in terms of body shape. This can lead to highly maladapted responses to selection. Hence, we can expect populations of organisms with highly integrated phenotypes to be more prevalent in scenarios with a lot of time for evolutionary adaptation. On the other hand, we can expect populations adapting to highly fluctuating environments in time—that is having less time for adaptation—to exhibit a lower level of integration. This lower level of integration would correspond to *smaller* regulatory networks in our simulation study (see Figure 6).

### 4.4. Evolution of Prochlorococcus

Comparative genomic studies for different species of the most abundant photosynthetic organism, *Prochlorococcus*, revealed that during speciation genome sizes of these organisms had considerably shrunk [36]. During speciation *two* effects occur simultaneously; on the one hand, species become more specialized and adapt to specific niches. Within such an ecological niche, environmental heterogeneity is decreased. At the same time, competition is increased before the evolution of specialized species is complete. This in turn leads to an increased time pressure of adaptation, that is, less time for evolutionary adaptation. Both factors lead to a preference to small regulatory networks, as observed for the genome sizes of these species during their evolution. This preference is also resulting from our simulation model, for this example involving both change in environmental heterogeneity and time pressure of evolutionary adaptation (see Figure 6).

As to discuss benefits and constraints of the conceptual approach chosen in our simulation study, we refer to Wissel [37] and Shubik [38] who call for the *parsimonious* modeling approach—even when dealing with apparently complex systems such as biological regulatory networks. Also, Lenski et al. [39] conclude that, studying digital organisms, that is, simplified models of regulatory systems, offers a useful tool for addressing biological questions in which complexity is both a barrier to understanding and an essential feature of the system under study. In our case, the structure of the Steinbuch matrix model [22] is that of the so-called *multi-input motif* which was found to be systematically enriched in molecular networks of prokaryotes as well as eukaryotes [23, 24]. The Boolean logic modeling biological regulatory interactions have been introduced and discussed, for example, by Kauffman [7] as well as by Somogyi and Sniegoski [8]. Nolfi and Parisi [40] described an approach to evolve neural networks, and discussed the genotype-phenotype mapping for their case—inspiring our own approach of evolving simple models of regulatory networks. Also, Frank [13] analyzed the population and quantitative genetics of evolving Boolean regulatory networks, and evaluated the performance as well as the effects of mutations in regulatory networks of different *connectivity*, while our studies were concentrated on the *size of regulatory networks*. Our study aims in the same direction of investigating system properties of a new synthesis of the population genetics of development, using explicit modeling of the genotype-phenotype-map, as called for by Johnson and Porter [41].

Interrelations of *network size* with evolutionary adaptation processes—even within our simulation study—were difficult to assess, as variation of mean population fitnesses was considerable between different runs. However, mean tendencies, as observed in our study for thousands of replicate runs with different randomly generated environmental challenges and target adaptations were *significant*. We conclude that for scales of evolutionary adaptation the observed tendencies are, hence, also *relevant* constraints.

In our modeling approach, environmental heterogeneity is simulated as sets of randomly drawn input vectors to the simulated regulatory networks. Here, the size, , of such an input set corresponds to the environmental heterogeneity. Environmental heterogeneity is a major determinant of an organisms fitness as it requires a minimum of adaptability, either on the phenotypical or on the genetical level [20]. On the phenotypical level, our modeling approach simulates adaptability through allowing an individual regulatory network to differentially respond to a number of different inputs, while its genetics—determining the *wiring* of the regulatory network—remains fixed. On the genetical level this *wiring* is subject to mutation and selection. There is, however, more towards possible structures of environmental input. As a possible extension of our study it would certainly be valuable to incorporate *long-term changes* within the environmental requirements. The set of input vectors may slowly change and demand a steady evolutionary adaptation. This change can occur on different time scales and with different degrees of autocorrelation. Here, we refer to the respective works on different noise colours as challenges in evolution [42, 43]. As a last possibly important parameter regarding model construction, we want to stress that the simulations did not take into account differences in *costs* for maintaining and running the networks. Adding this aspect would give extra evolutionary advantage to the smaller networks.

Summarising, the simplicity of our approach and model choice leads to very general predictions or explanations. However, it enables integrating over observations concerning regulatory structures from apparently distant disciplines and investigating common consequences of the structure of regulatory systems on a systems biology level. The main point of our contribution is the implementation of a special structure of parameter space (regulatory network encoding genotype) and the observation of the outcomes of a special sort of optimisation process (evolutionary dynamics for phenotype-based fitness function, where the phenotype is a function encoded by both genotype the regulatory network structure and environmental inputs). The results are interpreted on a system's biological background and linked to four biological examples which are very different concerning involved species, environments, and settings for individual adaptation and evolution, but structurally identical regarding our point of view. We consider our work as a small, hypothesis generating, contribution towards integrating findings of systems biological approaches concerning structure of biological regulatory networks with observations of their function regarding adaptability, result, and dynamics of adaptive evolution. Structures of regulatory modules within living organisms are on the one side *constraints* for evolutionary adaptation. On the other side, these structures themselves are adapted to heterogeneity of environmental variation, leading to optimized adaptability—as a compromise on both phenotypical and evolutionary levels. Further understanding of these interrelations will not only contribute to evolutionary biology, but also towards using and valuing genetic variation and adaptability in breeding programs of plant and livestock.
